# Menkes disease complicated by concurrent Koolen‐de Vries syndrome (17q21.31 deletion)

**DOI:** 10.1002/mgg3.829

**Published:** 2019-06-28

**Authors:** Taylor Woodfin, Christine Stoops, Joseph B. Philips, Edward Lose, Fady M. Mikhail, Anna Hurst

**Affiliations:** ^1^ Department of Pediatrics University of Alabama at Birmingham Birmingham Alabama; ^2^ Department of Genetics University of Alabama at Birmingham Birmingham Alabama

**Keywords:** Koolen‐de Vries, Menkes disease, overlapping phenotype

## Abstract

**Background:**

Koolen‐de Vries (KdV) syndrome is caused by a 17q21.31 deletion leading to clinical symptoms of hypotonia and developmental delay and can present with abnormal hair texture. Menkes disease is an X‐linked recessive inherited disease caused by pathogenic variants in *ATP7A*, which leads to profound copper deficiency.

**Method:**

We identified an infant male who presented with prematurity, hypotonia, and dysmorphic features for whom a family history of clinical Menkes disease was revealed after discussion with the clinical genetics team.

**Results:**

Although initial first‐tier genetic testing identified Kdv syndrome (17q21.31 syndrome), the family history led the team to consider a second diagnostic possibility, and testing of *ATP7A* revealed a pathogenic variant (c.601C>T, p.R201X).

**Conclusion:**

Menkes disease and KdV syndrome may both present with hypotonia and abnormal hair, in addition to seizures and failure to thrive. While these genetic conditions have overlapping clinical features, they have different natural histories and different therapeutic options. Here, we report on a patient affected with both disorders and review the diagnostic and therapeutic difficulties this presented.

## 1 INTRODUCTION

Dysmorphic findings in a newborn often warrant a genetic evaluation, emphasizing family history, clinical presentation, physical exam findings, as well as genetic testing. The importance of this complete approach is highlighted in a case where two rare conditions with overlapping clinical features coincide. The significance of this is further emphasized in that one of the conditions could potentially have an improved outcome if treatment is initiated early.

Koolen‐de Vries (KdV) syndrome (17q21.31 microdeletion; OMIM: 610443) is characterized by hypotonia, feeding difficulty, developmental delay (specifically gross motor and speech delay), seizures, pectus abnormalities, and characteristic facial features, which include a tubular nose with bulbous (“pear‐shaped”) tip, epicanthal folds, and abnormal hair texture. This syndrome occurs in 1 in 16,000 and the prognosis is highly variable (Koolen et al., 2008).

Menkes disease (OMIM: 309400) is an X‐linked recessive inherited disease that occurs in 1 in 100,000–300,000 live births (Kaler, 1994, 2011; Tønnesen, Kleijer, & Horn, 1991). Menkes disease is caused by pathogenic variants in *ATP7A* at Xq21.1, which encodes a transmembrane protein that mediates copper uptake from the intestine and delivers copper to the developing brain. Pathogenic variants lead to severe copper deficiency. Features include unusual “kinky” hair, growth retardation, bony abnormalities, profound neurodevelopmental delays, and seizures. The prognosis of untreated Menkes disease is poor; death often occurs in the first 3 years of life (Kaler, 1994). Daily subcutaneous administration of copper histidinate is the only current treatment and has been associated with variable outcomes depending on the age of initiation and *ATP7A* mutation type (Christodoulou et al., 1998; Kaler et al., 2008; Kaler, Tang, Donsante, & Kaneski, 2009; Sarkar, 1997; Tumer et al., 1996).

## 2 CASE PRESENTATION

Written informed consent was obtained from the parents of our patient. We report a boy born at 35.5 weeks gestation to a 36‐year‐old female with one prior spontaneous abortion. He was born via Cesarean section due to preterm labor and breech presentation. Pregnancy was complicated by maternal hypothyroidism, well‐controlled on medication. Cell‐free DNA screening in the first trimester was consistent with low risk for aneuploidy.

**Figure 1:**
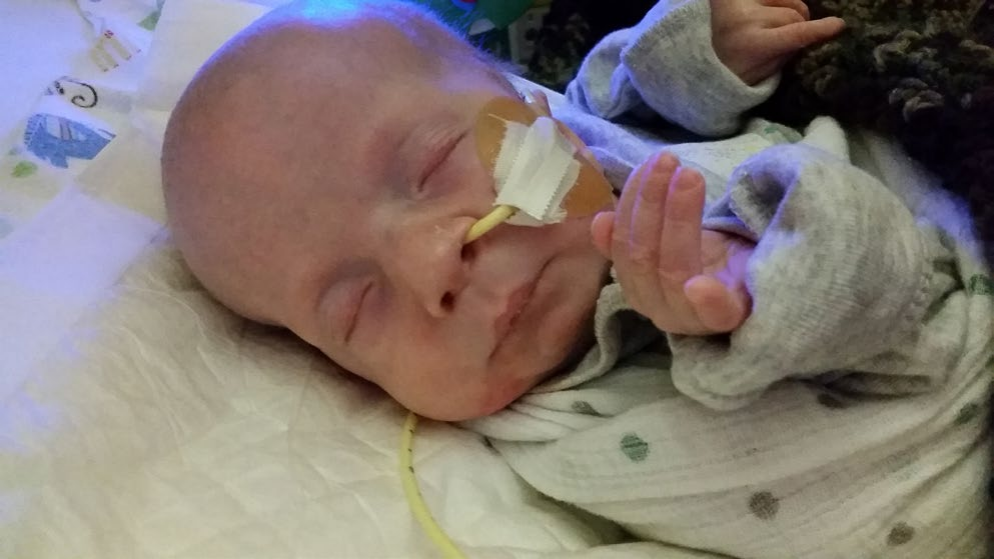
Photograph of the patient at 2 months of age

At birth, the patient weighed 2,275 g and length was 47 cm. He was noted to be hypotonic with dysmorphic features including low set ears, pectus excavatum, wide spaced nipples, cryptorchidism, and a broad, tubular nose with a bulbous tip (Figure 1). He was also noted to have sparse hair. A head ultrasound showed agenesis of the corpus callosum. He developed pulmonary hypertension soon after birth with increasing oxygen requirement, with echocardiogram showing normal anatomy and function, and was transferred to a tertiary hospital for inhaled nitric oxide therapy at 3 days of age.

**Figure 2:**
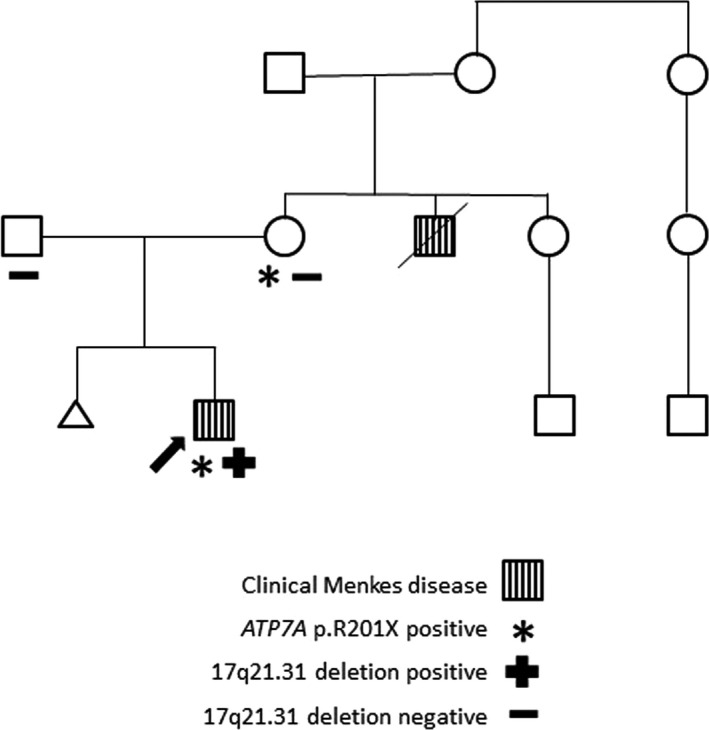
Pedigree demonstrating the proband (arrow) and his clinically affected maternal uncle. The proband and his mother tested positive for the *ATP7A* p.R201X variant, but no other family members sought testing. The proband was also positive for the 17q21.31 microdeletion, but his parents both tested negative

Medical genetics was consulted and obtained a complete family history including a maternal uncle who died at 13 months old due to congenital anomalies and diaphragmatic dysfunction (Figure 2). Family was told he had a clinical diagnosis of Menkes disease, although the diagnosis was not molecularly confirmed. Attempts to obtain records on the maternal uncle were unsuccessful.

Due to the hypotonia and dysmorphic findings, microarray comparative genomic hybridization (array CGH) was obtained. Molecular testing of *ATP7A* was ordered separately due to the family history; however, the latter was near the time of patient transfer to the second institution. When the outside molecular lab received the sample and contacted the initial hospital about billing, they were informed that the patient had been discharged and to cancel the *ATP7A* testing since it would not be reimbursed. The ordering provider was not informed of this decision.

The patient's pulmonary hypertension slowly improved after transfer without need for further escalation in management. His clinical course was complicated by significant feeding difficulties and profound hypotonia while awaiting test results.

**Figure 3:**
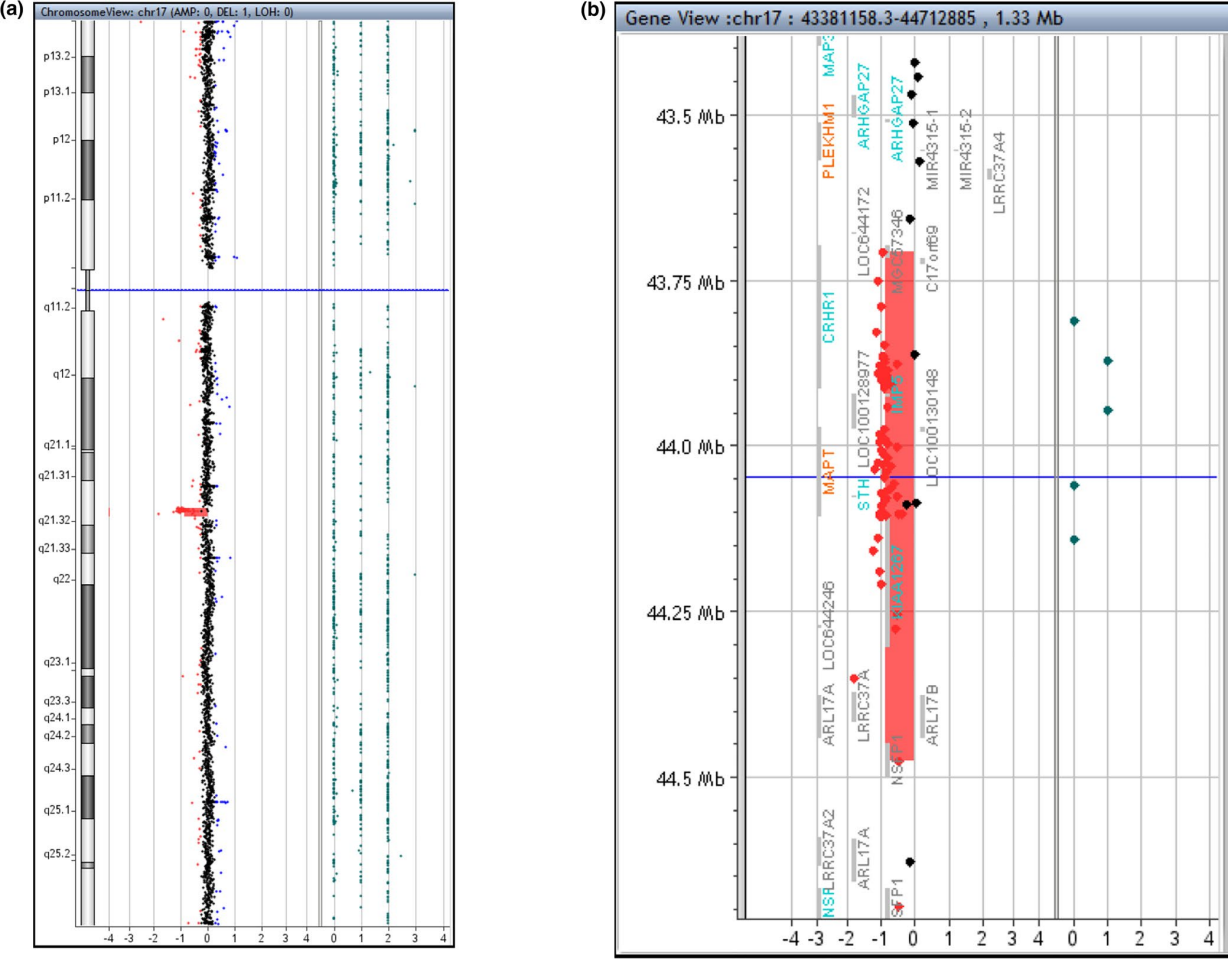
Array CGH analysis demonstrating the 17q21.31 microdeletion that spans the Koolen‐de Vries (KdV) syndrome typically deleted region. (a) Whole chromosome 17 view. (b) Zoomed‐in view demonstrating the 17q21.31 microdeletion (red shaded area)

Array CGH analysis using the Agilent 4x180k aCGH+SNP array (Agilent Technologies, Santa Clara, CA) showed a 501 kb deletion of 17q21.31 (arr[GRCh37]17q21.31(43,706,886‐44,208,036)x1), which is consistent with KdV syndrome (Figure 3). This was accepted as an explanation for his physical findings and clinical complications, including hypotonia, pectus excavatum, hair abnormalities, and dysmorphic findings.

At 5 weeks of life, the patient's clinicians contacted the outside molecular laboratory for an update on the status of *ATP7A* results and were informed of the initial institution's decision to cancel testing. Testing was rerequested, and revealed a hemizygous nonsense pathogenic variant in *ATP7A* (c.601C>T, p.R201X), which has previously been reported [9]. The *ATP7A* sequencing was performed at a CLIA ’88 compliant laboratory. PCR was used to amplify the 22 coding exons and immediate flanking regions of the *ATP7A* gene. The PCR products were sequenced in the forward and reverse directions.

In the interim, serum ceruloplasmin (<6 mg/dl) as well as his serum copper (8 μg/dl) was noted to be low. This represented a distinct genetic condition, unrelated to the previously diagnosed KdV syndrome. The diagnosis of Menkes disease was confirmed at just over 2 months of life in our patient.

Copper histidinate (FDA IND#34,166) is an experimental therapy for Menkes disease. Our patient was enrolled in a phase 3 clinical trial with the National Institutes of Health to obtain daily subcutaneous copper histidinate injections. These began at approximately 10 weeks of life.

The patient experienced clinical complications secondary to underlying pulmonary disease. Bronchoscopies were notable for significant tracheomalacia and bronchomalacia with a persistent oxygen requirement complicated by pulmonary hypertension, ultimately leading to tracheostomy placement with ventilatory support at 10 months of life. He achieved full oral feeds by 6 weeks of life (corrected 41 weeks of age), but experienced persistent feeding intolerance with emesis and evidence of laryngeal penetration; a gastrojejunostomy tube was placed at 5.5 months. He ultimately developed septic shock and died at 13 months of age from respiratory failure and pulmonary hypertensive crisis. The family declined autopsy.

Parental testing confirmed that the mother is a carrier for the *ATP7A* variant and neither parent carries the 17q21.31 microdeletion.

## 3 DISCUSSION

The neonatal symptoms of Menkes disease are nonspecific (e.g., prolonged jaundice, hypothermia) and usually not sufficient to suspect this rare condition. For this reason, diagnosis is often delayed until around 6–12 weeks of age or beyond, when more overt symptoms such as failure to thrive, hypotonia, and seizures present (Kaler, 1994). This highlights the importance of family history for early diagnosis and the need for newborn screening for this condition. For our patient, a greater appreciation of the suspected Menkes diagnosis in his maternal uncle may have reduced the time to diagnosis and enabled earlier initiation of copper histidinate treatment.

Most patients diagnosed with Menkes disease are found to have the severe classical form, including our patient who followed the expected natural history with limited life span [3]. The high mortality and neurocognitive decline are due not only to low copper levels but also because of the various enzymes dependent on copper as a cofactor. These include cytochrome c oxidase, lysyl oxidase, dopamine ß‐hydroxylase, peptidylglycine α‐amidating monooxygenase, superoxide dismutase, and tyrosinase (Kaler, 1994, 2011).

Copper histidinate injections continue to represent the most promising treatment for Menkes disease. Many studies have shown some benefit in delaying the progression of neurocognitive decline with copper histidinate injections, especially when administered early in life (Christodoulou et al., 1998; Kaler, 2014; Kaler et al., 2008, Kaler et al., 2009; ; Kim et al., 2014; Sarkar, 1997; Sparaco, Hirano, Hirano, DiMauro, & Bonilla, 1993; Tümer, 2013; Tümer & Møller, 2010; Tumer et al., 1996). A patient with the same *ATP7A* nonsense mutation (R201X) as found in our patient received copper histidinate subcutaneous injections starting at 8 days of life and had an excellent therapeutic response with essentially no neurocognitive deficits (Kaler et al., 2009). He is now 22 years old, completed high school and takes college classes, has a driver's license, and is a registered voter [personal communication]. This successful long‐term outcome in an individual with an *ATP7A* nonsense mutation supports the paramount importance of early diagnosis and treatment. In general, the best clinical outcomes with copper histidinate therapy in Menkes disease are dependent on early initiation plus the presence of partial ATP7A activity (Kaler, 2014); accordingly, viral gene therapy to restore working copies of *ATP7A* in combination with subcutaneous copper histidinate injections is currently in development (Haddad et al., 2018).

Prognosis in KdV syndrome (17q21.31 microdeletion) remains poorly defined due to lack of long‐term studies, recent description, and wide clinical variability. Notably, the diagnosis usually carries a prognosis that is in stark contrast to Menkes disease. While Menkes disease typically has a high mortality rate, KdV syndrome has variable outcomes depending on presentation. At this time, KdV syndrome is believed to be underrecognized in the intellectual disability population. The 17q21.31 microdeletion syndrome was first identified after screening of large heterogeneous cohorts of individuals with intellectual disability using high‐resolution chromosomal microarray screening technologies (Koolen et al., 2008).

The overlapping clinical features of the two distinct conditions in our patient likely contributed to a delayed diagnosis of Menkes disease. The most notable overlapping features include hypotonia, abnormal hair, pectus abnormalities, and seizures. Hypotonia is typically present at birth in KdV syndrome; however, in Menkes disease, it typically presents at 6–12 weeks of age or later (Kaler, 1994).

There are several case reports describing patients known to have KdV syndrome who clinically have abnormal hair texture or color (Koolen et al., 2012; Tan et al., 2009; Zollino et al., 2012). One case series described the hair as fine, sparse, and depigmented. One case in this series even showed a patient whose hair “kinked” when pressured by a glass slide. This case series also describes skin findings in several patients with KdV syndrome (Tan et al., 2009). As noted above, hair and skin findings are sometimes the first clinical findings seen in Menkes disease, so this overlap created a complicated clinical presentation in our patient.

The prospect of multiple genetic diagnoses in a single patient has been studied previously. A retrospective analysis of patients with a known molecular diagnosis involving Mendelian disease gene related to the clinical phenotype showing that 4.9% of these patients actually had two to four diagnoses (Posey et al., 2017). The authors concluded that a diagnostic evaluation is not necessarily complete with the identification of an initial molecular diagnosis, and genome‐wide analyses may reveal more than one Mendelian disease that is relevant for a patient and the patient's family (Posey et al., 2017). This theoretical possibility is especially significant in this example, where two diagnoses with overlapping clinical phenotypes coincided.

This case also highlights the value of obtaining a thorough family history. Without the family history, Menkes disease may not have been considered in the differential diagnosis, especially after the pathogenic array CGH finding for KdV. This case also serves to caution against premature closure of diagnostic inquiries. Had this patient only received the diagnosis of KdV, he would not have received a therapy with potential to improve his clinical course.

## 4 CONCLUSION

This case highlights the importance of a thorough evaluation and avoidance of premature closure. Our patient had two rare conditions with very different natural histories and treatment courses, but many overlapping clinical features.

## CONFLICTS OF INTEREST

The authors declare that there are no conflicts of interest regarding the publication of this paper.
